# Rapid Mercury(II) Removal by Electrospun Sulfur Copolymers

**DOI:** 10.3390/polym8070266

**Published:** 2016-07-20

**Authors:** Michael W. Thielke, Lindsey A. Bultema, Daniel D. Brauer, Bernadette Richter, Markus Fischer, Patrick Theato

**Affiliations:** 1 Institute of Technical and Macromolecular Chemistry, University of Hamburg, Bundesstr. 45, 20146 Hamburg, Germany; Michael.Thielke@chemie.uni-hamburg.de (M.W.T.); Lindsey.Bultema@chemie.uni-hamburg.de (L.A.B.); Daniel.Brauer@chemie.uni-hamburg.de (D.D.B.); 2 Hamburg School of Food Science, University of Hamburg, Grindelallee 117, 20146 Hamburg, Germany; Bernadette.Richter@chemie.uni-hamburg.de (B.R.); Markus.Fischer@chemie.uni-hamburg.de (M.F.)

**Keywords:** electrospinning, inverse vulcanization, sulfur, mercury removal, polysulfide

## Abstract

Electrospinning was performed with a blend of commercially available poly(methyl methacrylate) (PMMA) and a sulfur-rich copolymer based on poly(sulfur-*statistical*-diisopropenylbenzene), which was synthesized via inverse vulcanization. The polysulfide backbone of sulfur-containing polymers is known to bind mercury from aqueous solutions and can be utilized for recycling water. Increasing the surface area by electrospinning can maximize the effect of binding mercury regarding the rate and maximum uptake. These fibers showed a mercury decrease of more than 98% after a few seconds and a maximum uptake of 440 mg of mercury per gram of electrospun fibers. These polymeric fibers represent a new class of efficient water filtering systems that show one of the highest and fastest mercury uptakes for electrospun fibers reported.

## 1. Introduction

Water pollution control is one of the current environmental problems demanding the urgent development of new types of materials for efficient, practical, and inexpensive ways to remove toxic agents, such as heavy metals. Particularly, mercury has aroused great concerns due to the high risks for environmental and human health [1]. Exposure to high levels of mercury causes severe, irreversible health issues, such as kidney damage, neurological disorders, and birth defects [2]. Mercury is released into aqueous environments in a number of ways, namely atmospheric deposition, erosion, and industrial discharge [3]. Current techniques for mercury removal from aqueous systems include chemical precipitation [4], ion-exchange [5,6], adsorption [7,8], and membrane filtration [9]. Of the available methods, adsorption stands out as a low-cost, simple process when compared to other procedures of mercury removal. However, current commercial adsorbents possess low binding affinities for mercury or low surface areas. For example, the most common commercially-utilized adsorbent is active carbon, which possesses an adsorption capacity of 12.38 mg/g [10]. To produce an ideal adsorbent, fabrication of a material with both high surface area and high affinity for mercury is required. An inexpensive and versatile technique for producing high surface area materials is electrospinning, which has received a persistent, growing interest over the last two decades due to its potential in a multitude of applications, including catalysis [11], energy storage [12], biosensing [13], drug delivery [14], and membranes [15]. Electrospinning produces polymer fibers from solutions with diameters ranging from several micrometers down to a few nanometers from a simple setup. Due the high surface-to-volume ratio (10–40 m^2^/g) of the resulting fibers, these electrospun fibers possess excellent properties for filter applications in membranes and exhibit high metal ion adsorption capacities compared to other common fibrous filtering systems [16,17,18]. Forming electrospun fibers results from entanglement of the macromolecules and can be influenced by the given parameters of the setup, such as the electric field or the conductivity of the solution. These parameters are specific for every polymer and its molar mass, which must exceed a certain critical molar mass specific to each polymer. Notably, polymers below the required molar mass may be blended with another polymer to form a homogenous polymer blend capable of forming electrospun fibers [19].

By employing electrospun blends it is possible to incorporate new mercury-binding materials in porous, high surface area mats. Recent reports have shown that sulfur copolymers feature a high affinity for mercury and thus, exhibit promising properties as materials for mercury removal [20,21]. Unlike well-known polymers with a carbon-based backbone, sulfur-containing polymers synthesized via inverse vulcanization contain oligosulfide chains as a backbone. As opposed to regular vulcanization, inverse vulcanization uses sulfur in a significant amount and reacts with small amounts of an unsaturated organic monomer such as oleylamine, 1,4-diphenylbutadiyne [22], diisopropenylbenzene [23], or limonene [20]. Therefore, sulfur can be used as an alternative feedstock for the synthesis of functional materials. The preparation of polymers directly from elemental sulfur is a very promising approach for a new class of materials due to their unique properties and possible applications, such as cathode materials for lithium-sulfur batteries [22,24], infrared lenses with high refractive index [25], self-healing materials [26], and adsorbents for heavy metals [20,21]. Crucially, sulfur is produced in excess as a byproduct of the petroleum industry, making it relatively inexpensive and accessible. Poly(sulfur-*statistical*-diisopropenylbenzene) (poly(SDIB)) was the first sulfur copolymer reported that was synthesized via inverse vulcanization, and has been investigated for these different purposes. Although the resulting materials have different properties from carbon-based polymers, these sulfur polymers can be processed with similar methods, making them viable candidates for electrospinning. Utilizing the poly(SDIB) for electrospinning is a new approach for maximizing the surface properties of this material. Unlike other electrospun fibers, these polymeric fibers use the reactive backbone for the adsorption of heavy metals. By combining the high surface-to-volume ratio of electrospun fibers and the high affinity towards heavy metals of the poly(SDIB), these systems present a new and promising way for heavy metal removal.

## 2. Materials and Methods

All chemicals were commercially available and used as received unless otherwise stated. Tetrahydrofuran (THF) and dimethylformamide (DMF), both p.a. grade, were purchased from VWR Chemicals (Darmstadt, Germany), sulfur was purchased from Carl Roth GmbH and Co. KG (Karlsruhe, Germany). Diisopropenylbenzene (DIB) was purchased from TCI chemicals (Eschborn, Germany). *N*-benzylmethylamine, mercury(II) chloride, cadmium nitrate tetrahydrate, cobalt(II) nitrate hexahydrate, zinc nitrate hexahydrate, and lead(II) nitrate were purchased from Sigma Aldrich (Munich, Germany). Nitric acid (65%) was purchased from Th. Geyer GmbH and Co. KG. (Hamburg, Germany). Copper(II) nitrate trihydrate, l-cysteine, and Iron(III) nitrate nonahydrate were purchased from Merck KGaA (Darmstadt, Germany), and Poly(methyl methacrylate) (PMMA) (*M*_w_ = 109,000 g/mol, *M*_w_/*M*_n_ = 1.86) was gratefully granted by Evonik Industries AG (Essen, Germany).

### 2.1. Synthesis of Poly(SDIB)

**Scheme 1:**
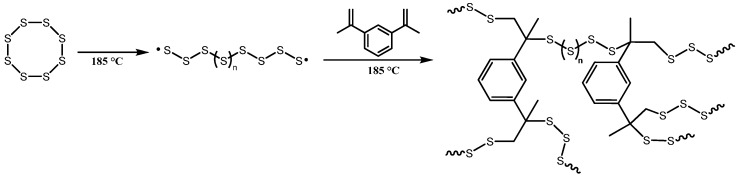
Synthetic route for the inverse vulcanization of sulfur and diisopropenylbenzene yielding poly(sulfur-*statistical*-diisopropenylbenzene). Sulfur polymerizes via ring-opening polymerization and the formed polysulfide radicals subsequently react with the diisopropenylbenzene via inverse vulcanization.

Poly(sulfur-*statistical*-diisopropenylbenzene) was synthesized as previously described in the literature [23] and shown in Scheme 1. The synthesis was performed on a 3 g scale according to the literature with 50 wt % of sulfur, 49 wt % diisopropenylbenzene, and 1 wt % *N*-benzylmethylamine. The mixture was stirred at 185 °C for five min to initiate the ring-opening polymerization of sulfur. Completion was indicated by a change in color of molten sulfur from clear yellow to dark red and complete vitrification of the solution. The resultant solution was cooled to room temperature and yielded 3 g of a transparent red polymeric glass. The complete conversion of sulfur to poly(SDIB) was verified via dynamic scanning calorimetry (DSC) at a rate of 10 K/min (Supporting Information, Figure S1). The measurement proved the absence of sulfur due to the missing endothermic signal, which was caused by the melting of sulfur at 115 °C. The comparison of the ^1^H-NMR of poly(SDIB) (Supporting Information, Figure S2) and the diisopropenylbenzene (DIB) showed an immense decrease in detected double bonds. 

### 2.2. Analytical Methods

Inductively-coupled plasma mass spectrometry (ICP-MS) was performed on an Agilent Technologies 7700× series mass spectrometer (Agilent Technologies, Santa Clara, CA, USA). Scanning electron microscopy (SEM) images were taken with an EVO-MA 10 microscope (Zeiss, Jena, Germany).operating at 5 kV. Nuclear magnetic resonance (NMR) spectroscopy was measured on a Avance 400 MHz Spectrometer (Bruker, Bremen, Germany) using tetramethylsilane (TMS; δ = 0 ppm) as the internal standard. The heating curves were measured by differential scanning calorimetry (DSC) using a DSC 1 Stare System (Mettler Toledo, Gießen, Germany) at a heating rate of 10 K·min^−1^.

### 2.3. Electrospinning

Electrospinning was performed with a standard horizontally-aligned setup using a mixed polymer solution composed of 15 wt % poly(SDIB) and 7.5 wt % PMMA, dissolved in a mixture of THF/DMF 7:3 (*w/w*). The applied voltage was set to 20 kV and a syringe pump was used with a blunt metal needle (0.8 mm diameter) at a flow rate of 1 mL/h and a tip-to-collector distance of 15 cm. The fibers were collected on an aluminum substrate placed on the grounded collector.

### 2.4. Metal Ion Sorption

For Hg(II) sorption studies, 20 mL aqueous HgCl_2_ solutions of various concentrations (0.1, 0.7, and 3.0 mM) were added to a glass vial containing 20 mg of poly(SDIB)/PMMA fibers (1 mg/mL). The solution was stirred at room temperature for 60 min and aliquots of 1 mL were removed at different time points between five seconds and one hour. The samples were filtered through a polyethylene terephthalate (PET) syringe filter with a pore size of 1.2 µm, diluted by factors of 1000 to 100,000, and subjected to ICP-MS measurements. For additional metal ion samples (cadmium nitrate tetrahydrate, cobalt(II) nitrate hexahydrate, zinc nitrate hexahydrate, lead(II) nitrate, copper(II) nitrate trihydrate, and iron(III) nitrate nonahydrate) 10 mL of a 0.7 mM aqueous solution was added to a glass vial containing 10 mg of poly(SDIB)/PMMA fibers (1 mg/mL). The solution was stirred at room temperature and a sample of 1 mL was removed after 60 min. The sample was filtered through a PET syringe filter with a pore size of 1.2 µm, diluted by a factor of 100,000 and subjected to ICP-MS measurements.

## 3. Results

### 3.1. Electrospinning

Polymer fiber mats were fabricated containing poly(sulfur-*statistical*-diisopropenylbenzene) (poly(SDIB)), which was synthesized via inverse vulcanization with a sulfur content of 50 wt % as reported in the literature [15]. First tests on electrospinning pure poly(SDIB) solutions in THF/DMF showed unsatisfying results, such as a broad fiber diameter, very heavy beading and melting together at the contact points of different fibers due to the low glass transition temperature (26.6 °C) of the poly(SDIB) (Supporting Information, Figure S3). Therefore, a mixed solution of poly(SDIB) and high molecular weight PMMA (*M*_w_ = 109,000 g/mol, *M*_w_/*M*_n_ = 1.86) was used to improve the resulting mechanical properties of the fibers. The PMMA/poly(SDIB) blend produced thinner, evenly-formed fibers without fused areas, thus improving the mechanical properties. PMMA is well-known for being used for electrospinning, due to high solubility in organic solvents, while remaining insoluble in aqueous solutions. The solution was selected to fabricate smooth fibers with a high amount of poly(SDIB). Therefore, the solutions were made with 15 wt % of poly(SDIB), resulting in an almost fully-saturated solution. Poly(SDIB) was dissolved in various PMMA solutions in THF/DMF 7:3 (*w/w*), starting from 3 wt % of PMMA . The amount of PMMA was increased until the electrospinning resulted in smooth fibers, as seen in Table 1 (SEM images in Supporting Information, Figures S4–S9). The best results with consistently-sized fibers and a high amount of poly(SDIB) were achieved by using a mixture of 15 wt % of poly(SDIB)and 7.65 wt % of PMMA, dissolved in THF/DMF 7:3 (*w/w*).

**Figure 1:**
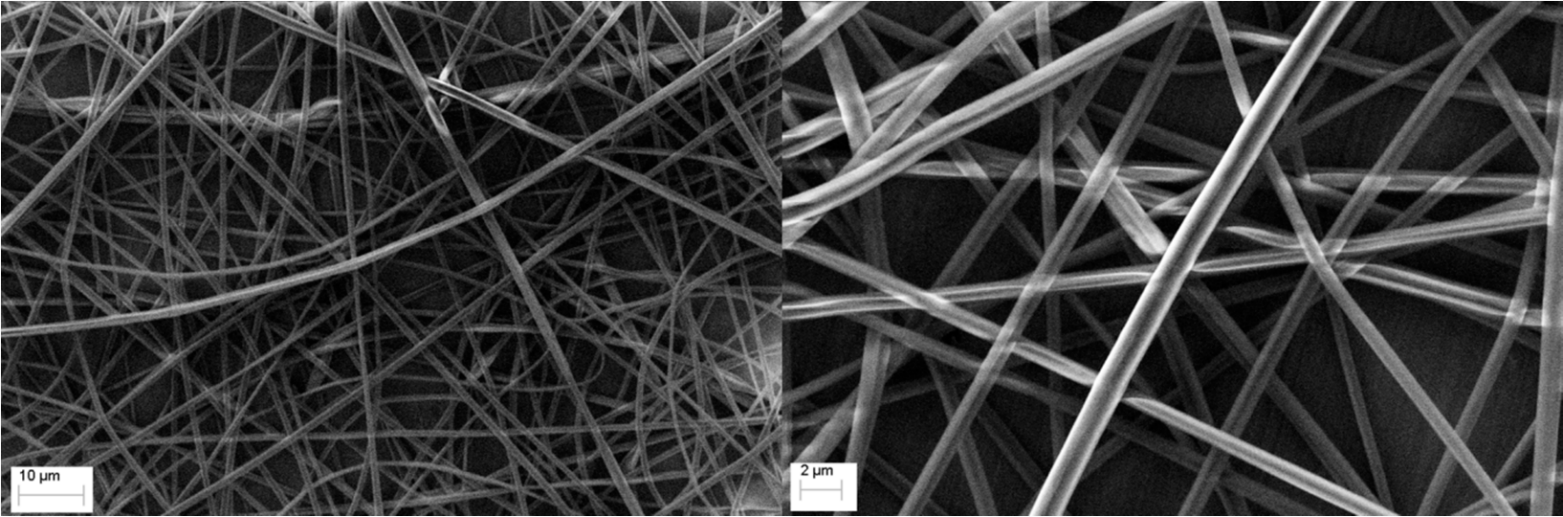
Scanning electron microscopy images of the electrospun poly(SDIB)/PMMA fibers. The average diameter of the fibers was determined to be 0.970 ± 0.220 µm.

The composition of the resulting fibers was 66.2 wt % of poly(SDIB) and 33.8 wt % of PMMA, assuming the complete evaporation of the solvent. Reducing the amount of PMMA led to beading within the fiber and increased poly(SDIB) content was not possible due to solubility issues. The average diameter of the fibers was determined to be 0.970 ± 0.220 µm through the analysis of the SEM-images (Figure 1).

The surface free energy (SFE) of the polymers was determined via Young's equation using contact angles of water and formamide [27]. PMMA, poly(SDIB), and the blended solution were spin-coated onto glass substrates at 8000 rpm from a solution in THF/DMF 7:3 (*w/w*), which is also the solvent in the electrospinning process. The results are listed in Table 2.

The SFE of PMMA and poly(SDIB) were determined to be 38.8 and 27.4 mN/m, respectively. Therefore, the SFE of poly(SDIB) is substantially lower, which favors poly(SDIB) exposure on the surface. The SFE of the blended film was determined with 30.1 mN/m, which is very close to pure poly(SDIB).

### 3.2. Metal Ion Sorption Studies

To determine the effectiveness of the electrospun fibers on the removal of Hg(II) from water, 20 mg (total of 0.32 mmol sulfur) of poly(SDIB)/PMMA fibers and 20 mL of a HgCl_2_ solution (0.1, 0.7, or 3.0 mM) were stirred for up to 60 min. The adsorbed Hg(II) was determined by measuring the remaining Hg(II) in solution through ICP-MS, with standard concentrations of HgCl_2_ ranging from 0 to 600 ppm, including nitric acid (2%) and l-cysteine (0.18 M). The freshly-prepared standards and samples were diluted by factors of 1000 to 100,000. The affinity of the fibers to remove Hg(II) from water was characterized by the distribution coefficient (*K*_d_) [28]. *K*_d_ is defined as:

(1) $$K_{d}=\frac{\left(C_{i}-C_{f}\right)}{C_{f}}\times \frac{V}{m}$$

where *C*_i_ is the initial metal ion concentration (mg/L), *C_f_* is the equilibrium metal ion concentration (mg/L), *V* is the volume of the solution (mL), and *m* is the mass of poly(SDIB) fibers used (g). The *K*_d_ demonstrates any sorbent’s performance metrics of metal ion adsorption. *K*_d_ values of 1.0 × 10^5^ mL/g and above are considered excellent [29]. The *K*_d_ values of the poly(SDIB) fibers for Hg(II) were determined to be 3.94 × 10^4^, 1.42 × 10^4^, and 2.71 × 10^3^ mL/g for the 20, 140, and 600 ppm solutions, respectively. These values are within the range of previously-described materials for Hg(II) removal [30,31,32,33,34,35].

**Figure 2:**
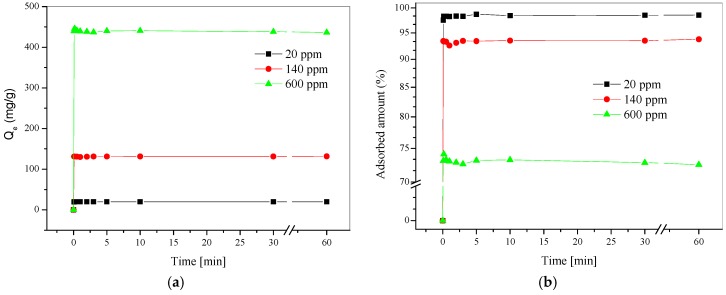
(**a**) PPM of Hg(II) adsorbed to the poly(SDIB)/PMMA fibers versus the time of poly(SDIB)/PMMA fibers in HgCl_2_ solution. (**b**) Adsorbed percentage of Hg(II) versus the time of fibers in HgCl_2_ solution.

The mercury uptake capacity of poly(SDIB)/PMMA fibers was calculated by the mass balance equation:

(2) $$Q_{e}=\frac{\left(C_{o}-C_{e}\right)V}{m}$$

where *Q*_e_ is the adsorption capacity (mg/g), *C*_o_ is the initial concentration of Hg(II) in solution, *C*_e_ is the final concentration of Hg(II) in solution, *V* is the volume of solution (mL), and *m* is the mass of poly(SDIB) fibers (g). As shown in Figure 2a, the maximum mercury adsorption of the poly(SDIB)/PMMA fibers was calculated to be for 19.7, 125.9, 327.7 mg/g for 20, 140, and 600 ppm, respectively.

Additionally, the percentage of mercury removed from the solution was calculated with the following equation:

(3) $$R=\frac{C_{o}-C_{t}}{C_{o}}\times 100\%$$

where *R* is the percentage of Hg(II) removed, *C*_o_ is the initial concentration of Hg(II) in solution, and *C_t_* is the concentration of Hg(II) at a specific time (mg/L) (Figure 2b). As shown in Figure 2b, the poly(SDIB)/PMMA fibers rapidly captured Hg(II) ions for the lowest concentrated starting solution (20 ppm) and after a few seconds more than 98.3% of the Hg(II) was removed; whereas for the higher concentration (600 ppm) roughly 73.1% adsorption was observed. This suggests a high adsorption capacity and high adsorption affinity for the removal of Hg(II) from water.

The mercury uptake can be attributed to the high surface area that is characteristic for electrospun fibers. The rate of adsorption rapidly transitions to a gradual adsorption and reaches the equilibrium adsorption capacity. The high initial adsorption rate may be attributed to the available polysulfide backbone with vacant adsorption sites. As time progressed there was a decrease in the available adsorption sites which, in turn, resulted in lowering the adsorption rate. Subsequently, upon reaching saturation of the poly(SDIB) fibers, the amount of adsorbed Hg(II) remained constant. The pH-value of the aqueous phase was determined before and after the absorption study. A significant decrease in pH was observed for each of the HgCl concentrations, from a pH of 6 to 4, 5 to 2, and 5 to 3 for 20 ppm, 140 ppm, and 600 ppm, respectively. The decrease in pH is due to the release of chloride. Additionally, the absorbance for other metal ions was investigated. The adsorption was performed with a 0.7 mM solution for one hour and the extant concentration was determined by ICP-MS. The results are listed in Table 3.

**Figure 3:**
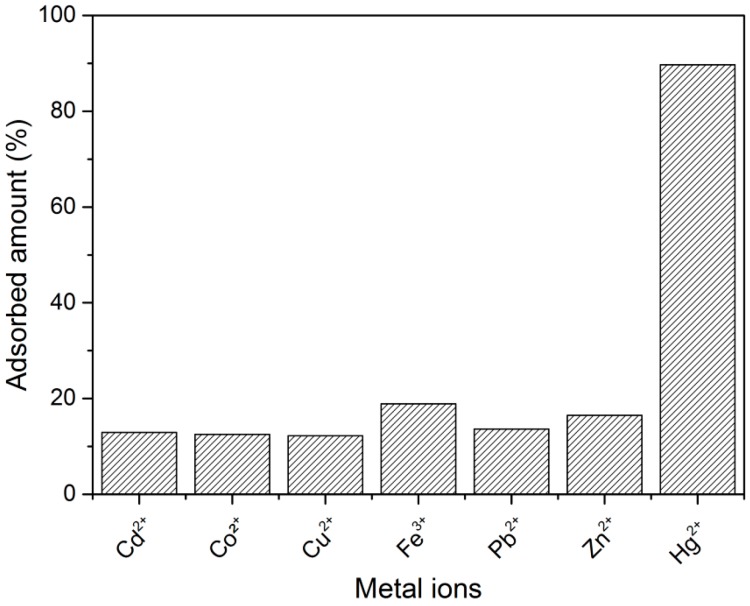
Comparison of the adsorbed percentage of metal ions after one hour of treatment with blended poly(SDIB)/PMMA fibers. Aqueous metal ion solutions were 0.7 mM and mixed with 1 mg material per mL solution.

The adsorbed percentages ranged from 12.2% to 18.9%, which was significantly lower than the observed Hg(II) adsorption of 93.3% The adsorbed percentage after one hour from different metal ion solutions (0.7 mM) is shown in Figure 3.

Additionally mercury(II) ion adsorption of thin films of the poly(SDIB)/PMMA was measured. The blend was spin-coated on a 1.8 cm^2^ glass substrate at 8000 rpm. A total of 35 films with a total weight of 13.2 mg and a total surface area of 113.4 cm^2^ were prepared and stirred in 13.2 mL of an aqueous 0.7 mM HgCl_2_ solution for one hour. The adsorbed percentage of mercury(II) was determined to be 3.3%. The minimal adsorption was attributed to the low surface area characteristic of thin films. Using the same mass of material, the electrospun fibers adsorbed more than 28 times the amount of mercury(II). The drastically-improved performance was attributed to the significantly greater surface area of the nanofibers, highlighting the importance of electrospun fibers for filtration applications.

## 4. Discussion

Herein, a method is described for the fabrication of electrospun fibers from a sulfur copolymer blended with commercially available PMMA. Due to their polysulfide backbone, sulfur copolymers, such as the used poly(SDIB), are able to remove heavy metals, such as mercury, from water. Therefore, poly(SDIB) is an outstanding candidate for water purification. To enhance the total surface area of the polymer and, thus, improve adsorption parameters, electrospun fibers were fabricated. Electrospinning of a mixture of PMMA and poly(SDIB) formed stable fibers with an average diameter of 0.970 ± 0.220 µm, which demonstrates a rapid and high uptake of mercury above any previously-reported fibers due to the presence of the polysulfide backbone. 

When considering a material that can be used in mercury removal from aqueous solutions, the distribution coefficient (*K*_d_) and adsorption capacity (*Q*_e_) are important parameters to consider for effective and efficient mercury removal. Herein, we have reported normal distribution coefficient and exceptional adsorption capacity values for the poly(SDIB) fibers. The poly(SDIB) fibers adsorbed between 70% and 98% of the Hg(II) ions, while the chloride accumulated in the aqueous solution, leading to a decreased pH value. The material displays an excellent adsorption capacity of 327.7 mg/g, which far exceeds the 12.38 mg/g adsorption capacity of commercially-available activated carbon [10]. The rapid removal of Hg(II) from the solution can be attributed the high surface area and polysulfide backbone of the polymer fibers. Additionally, we determined the adsorbed percentage of other metals to be between 12.2% and 18.9% after one hour in a 0.7 mM solution. To the best of our knowledge, this is the first report on the preparation of a stable sulfur copolymer containing fibers, which represents an important step in utilizing such materials in future filter and water purification systems.

## Figures and Tables

**Table 1:** Summary of conditions for electrospinning the poly(SDIB)/PMMA fibers with the resulting appearance, observed via SEM (Supporting Information, Figures S4–S9).

PMMA concentration (wt %)	Poly(SDIB) content of the fiber (wt %)	SEM-Resulting appearance
3	85.5	Few fibers, predominantly electrosprayed particles
4	81.5	heavily beaded fibers
5	77.9	heavily beaded fibers
6	74.6	beaded fibers
7	71.6	beaded fibers
8	68.8	predominantly smooth fibers, few beads
9 ^1^	66.2 ^1^	fibers ^1^

^1^ Used composition for the fabrication of the fibers.

**Table 2:** Determined surface free energy (SFE) of the used polymers and the blend.

Polymer	Concentration	SFE (mN/m)
PMMA	10 wt %	38.8 ± 0.9
Poly(SDIB)	10 wt %	27.4 ± 1.8
Blend	7.65 wt % (PMMA) + 15 wt % (Poly(SDIB))	30.1 ± 0.7

**Table 3:** Adsorbed percentage of other metal ions from electrospun fibers. The procedure was identical to the mercury(II) adsorption after 60 min.

Salt	Metal ion	Adsorbed percentage (%)
Cd(NO_3_)_2_·4 H_2_O	Cd^2+^	12.9
Co(NO_3_)_2_·6 H_2_O	Co^2+^	12.5
Cu(NO_3_)_2_·3 H_2_O	Cu^2+^	12.2
FeNO_3_)_2_·4 H_2_O	Fe^3+^	18.9
Pb(NO_3_)_2_	Pb^2+^	13.6
Zn(NO_3_)_2_·4 H_2_O	Zn^2+^	16.5

## Supplementary Materials

Supplementary Materials can be found at www.mdpi.com/2073-4360/8/7/266/s1.
