# Supplementary material for: Rapid Mercury(II) Removal by Electrospun Sulfur Copolymers
Source: Polymers (Basel). 2016 Jul 20;8(7):266. doi: 10.3390/polym8070266 (PMC6432393; doi:10.3390/polym8070266)
Supplement: Supplementary file 1 [file polymers-08-00266-s001.pdf]

Supplementary Materials: Rapid Mercury(II) Removal by Electrospun Sulfur Copolymers

Michael W. Thielke, Lindsey A. Bultema, Daniel D. Brauer, Bernadette Richter, Markus Fischer and Patrick Theato

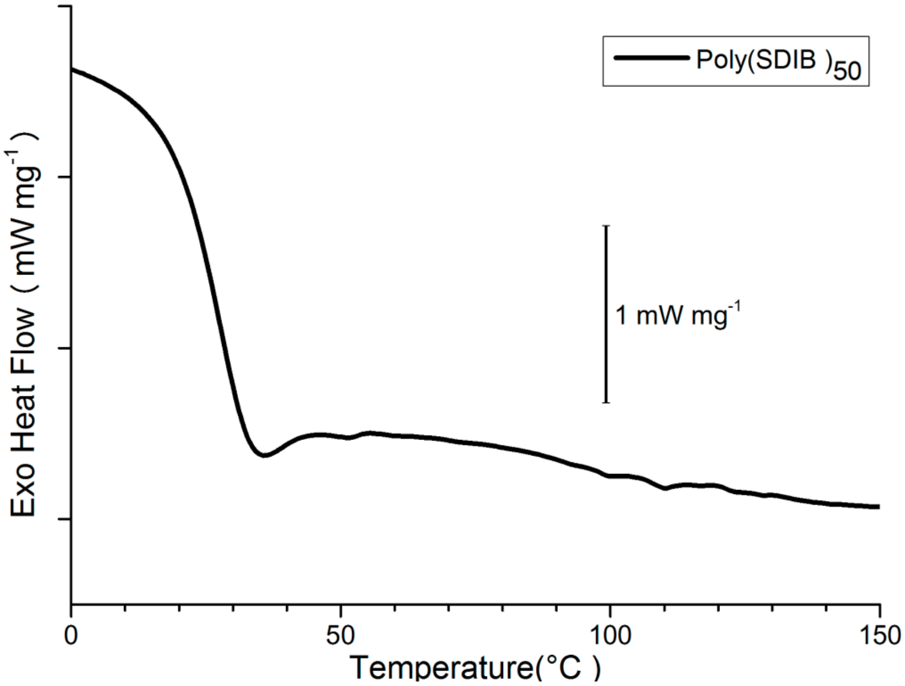

Figure S1. Dynamic scanning calorimetry measurement of the Poly(SDIB). The utilized sulfur was fully converted to Poly(SDIB) and showed no melting of S_8 at 115 °C.

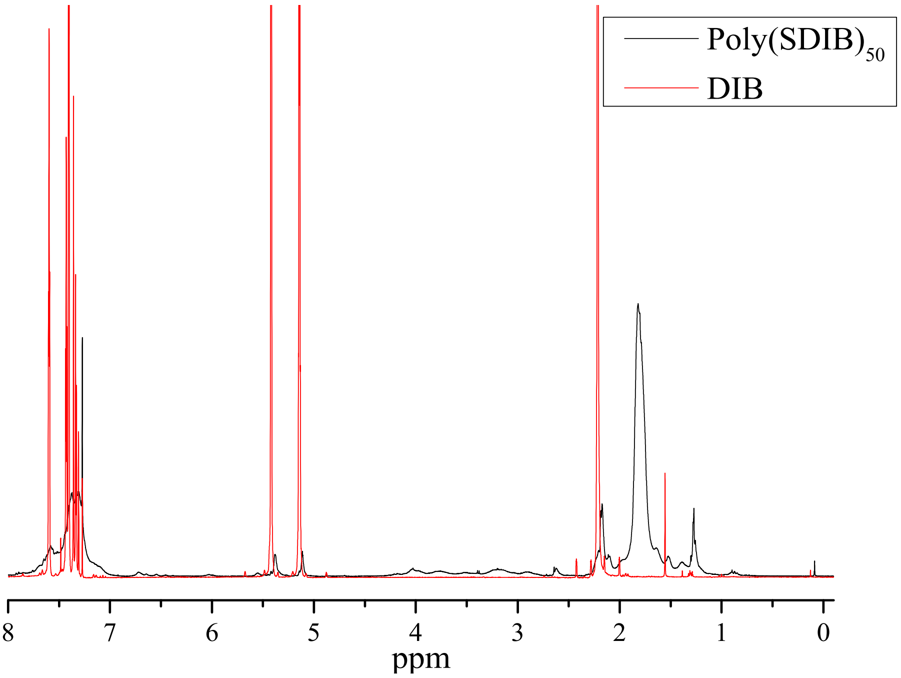

Figure S2. ^{1}H-NMR of Poly(SDIB)50 (black) and DIB (red). Compared to the DIB, the Poly(SDIB) shows a decrease of the signal at 2.2 ppm, while a new signal at 1.8 ppm arises. This is caused by reaction of the inverse vulcanization.

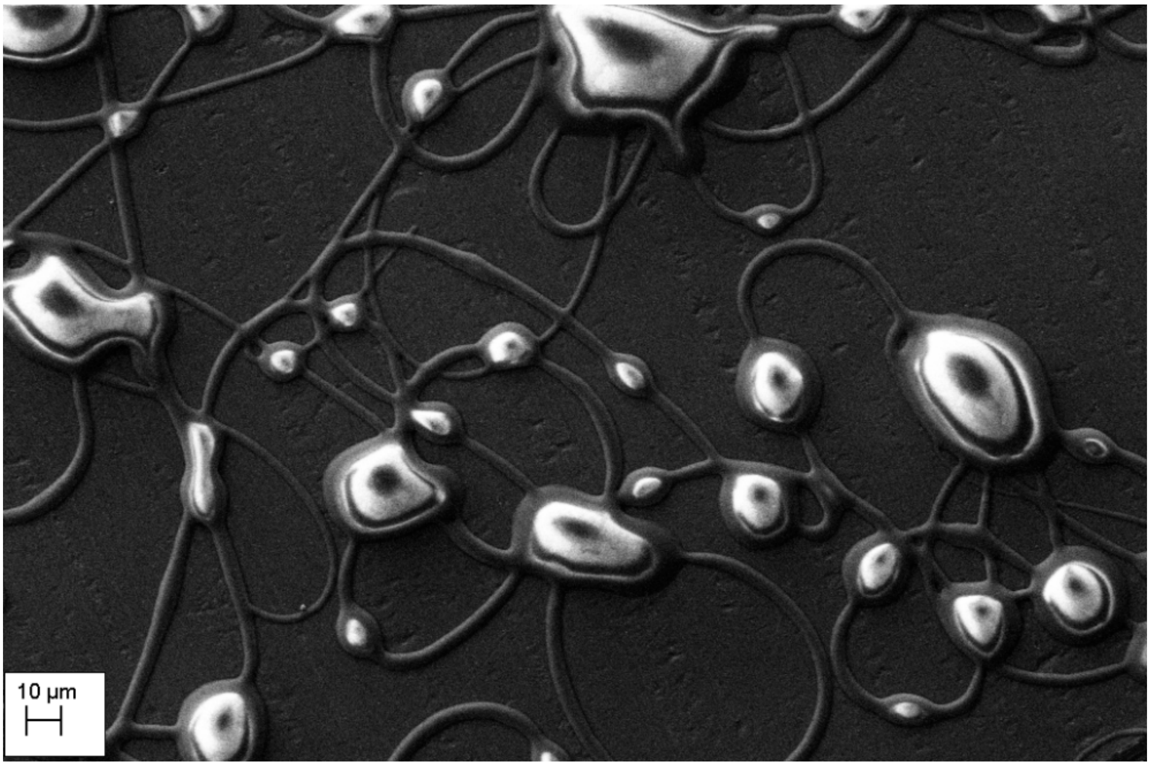

Figure S3. Scanning electron microscopy images of pure poly(SDIB) fibers from electrospinning. The resulting fibers melted to the collector surface and with each other on contact points.

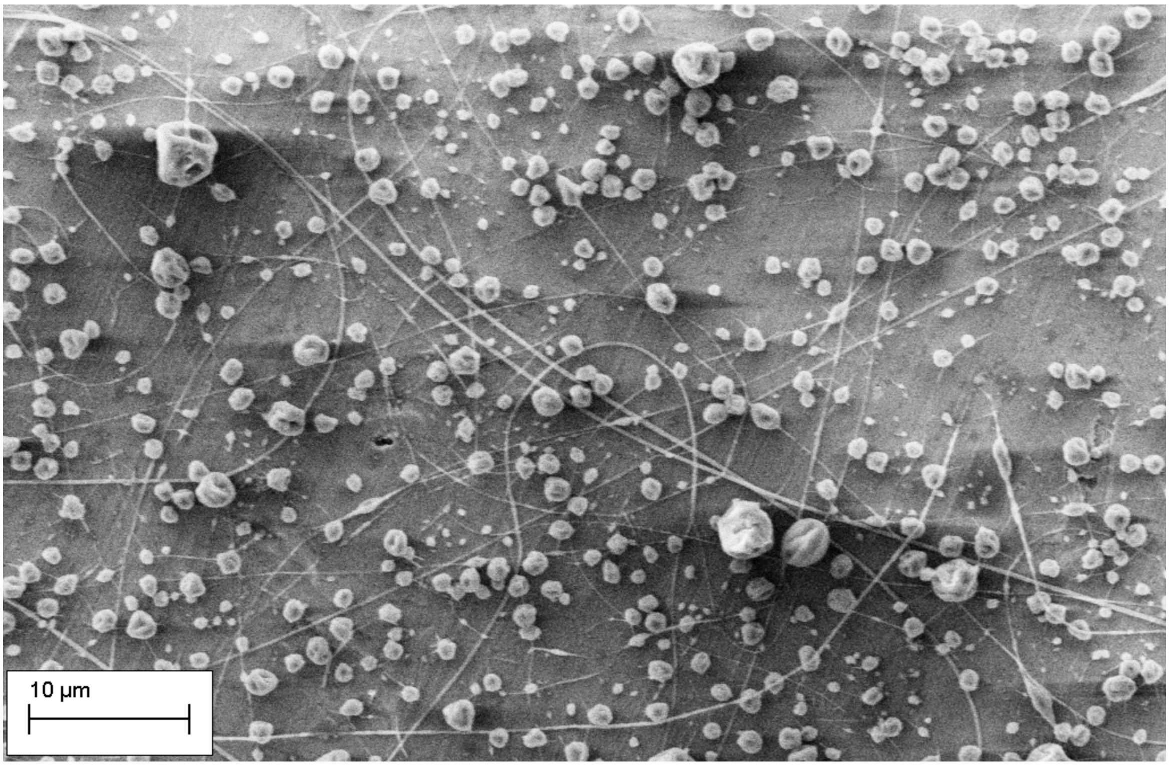

Figure S4. Scanning electron microscopy images of 15 wt % poly(SDIB) in a 3 wt % PMMA solution in THF/DMF (7:3; *w*:*w*).

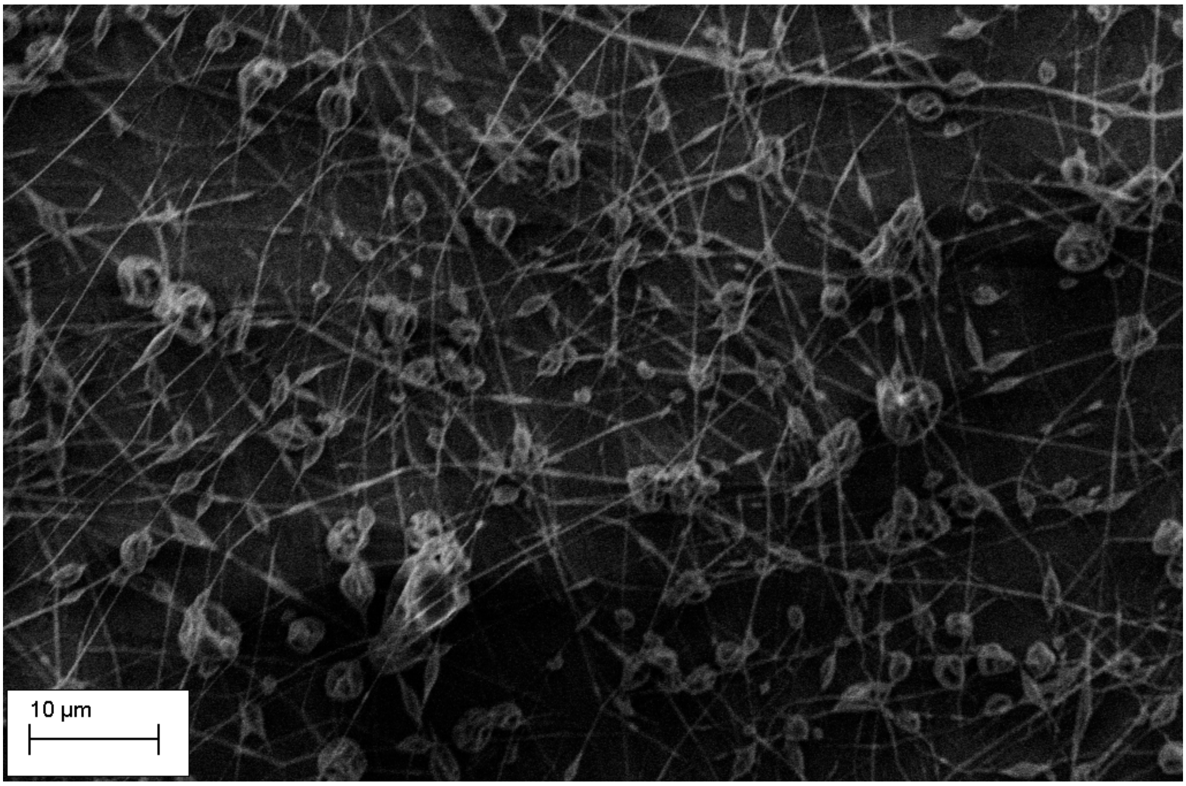

Figure S5. Scanning electron microscopy images of 15 wt % poly(SDIB) in a 4 wt % PMMA solution in THF/DMF (7:3; *w*:*w*).

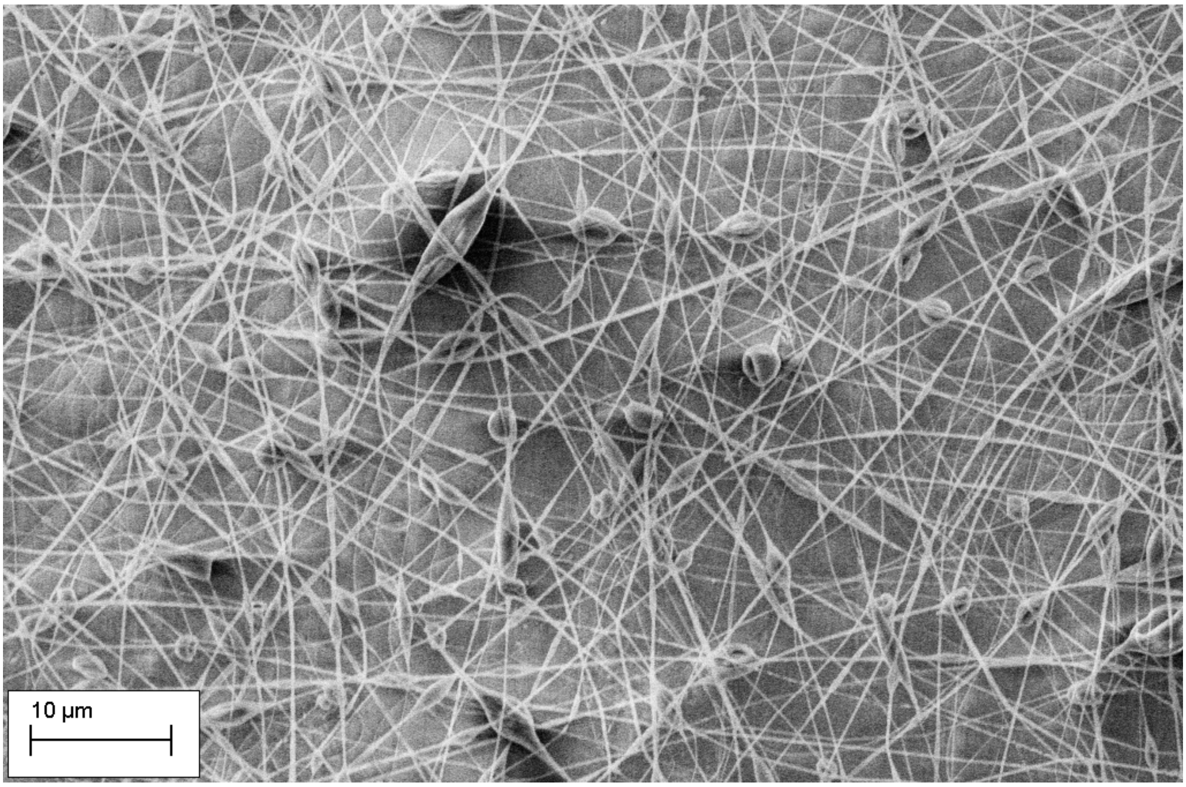

Figure S6. Scanning electron microscopy images of 15 wt % poly(SDIB) in a 5 wt % PMMA solution in THF/DMF (7:3; *w*:*w*).

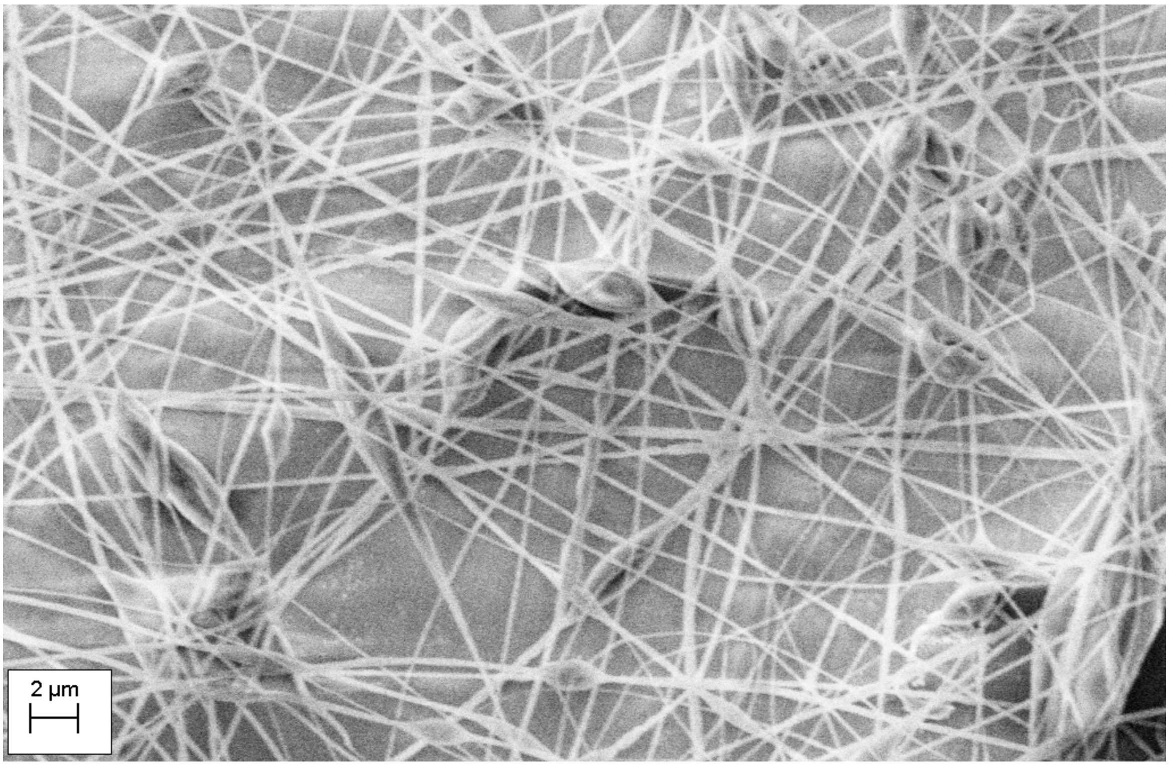

Figure S7. Scanning electron microscopy images of 15 wt % poly(SDIB) in a 6 wt % PMMA solution in THF/DMF (7:3; *w*:*w*).

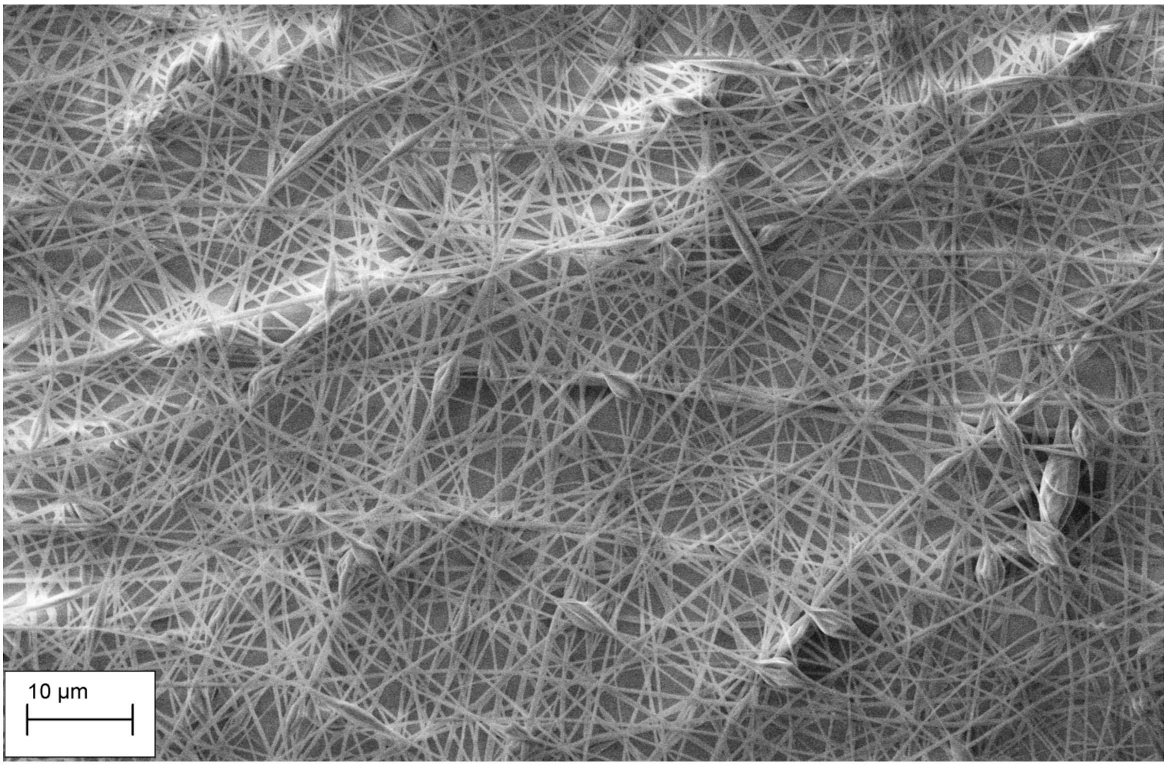

Figure S8. Scanning electron microscopy images of 15 wt % poly(SDIB) in a 7 wt % PMMA solution in THF/DMF (7:3; *w*:*w*).

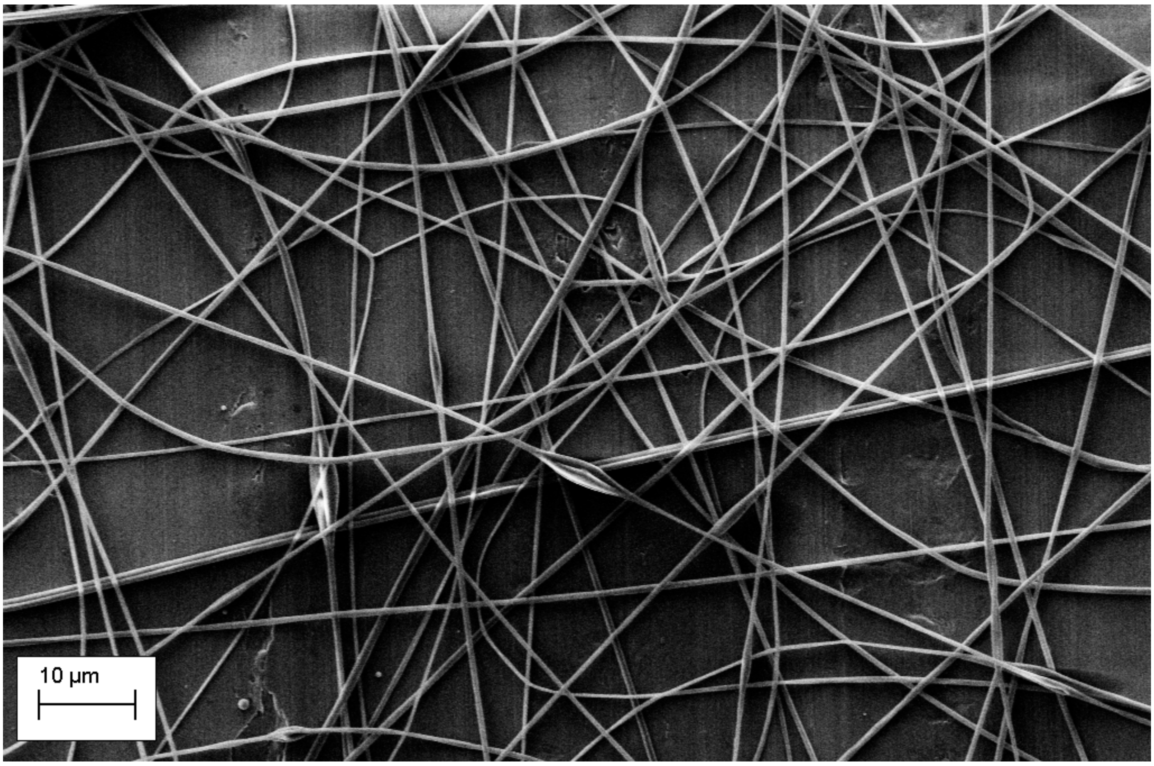

Figure S9. Scanning electron microscopy images of 15 wt % poly(SDIB) in a 8 wt % PMMA solution in THF/DMF (7:3; *w*:*w*).

© 2016 by the authors; licensee MDPI, Basel, Switzerland. This article is an open access article distributed under the terms and conditions of the Creative Commons Attribution (CC-BY) license (http://creativecommons.org/licenses/by/4.0/).
